# In vitro antioxidant and anti-inflammatory properties of *Artocarpus altilis* (Parkinson) Fosberg (seedless breadfruit) fruit pulp protein hydrolysates

**DOI:** 10.1038/s41598-023-28684-z

**Published:** 2023-01-27

**Authors:** Sodiq Oluwaseun Dada, Great Chimsom Ehie, Olukemi Adetutu Osukoya, Scholastica Onyebuchi Anadozie, Olusola Bolaji Adewale, Adenike Kuku

**Affiliations:** 1 Department of Chemical Sciences, Afe Babalola University, Ado-Ekiti, Nigeria; 2 Department of Biochemistry and Molecular Biology, Obafemi Awolowo University, Ile-Ife, Nigeria

**Keywords:** Biochemistry, Biological techniques

## Abstract

Protein hydrolysates from dietary sources possess many physiological and biological properties. *Artocarpus altilis* is an evergreen multipurpose plant with many benefits*.* Therefore, this study evaluates in vitro antioxidant and anti-inflammatory properties of *A. altilis* protein hydrolysates. Protein was isolated from *A. altilis* and hydrolysed with pepsin and trypsin separately using different enzyme: substrate ratios (1:8, 1:16, 1:32). Antioxidant properties investigated included Fe^2+^-chelating, 2,2-diphenyl-1-picrylhydrazyl (DPPH) radical and hydrogen peroxide radical scavenging activities. Anti-inflammatory activities were determined using effects on hypotonic solution-induced cell lysis on red blood cell membrane stabilisation and heat-induced protein denaturation. The degree of hydrolysis of trypsin hydrolysate increased with increasing enzyme–substrate ratio, while pepsin hydrolysate decreased as the enzyme–substrate ratio increased. The dominant amino acids in *A. altilis* protein and hydrolysates were glutamate, aspartate and leucine. Protein hydrolysates obtained from pepsin and trypsin digestion had DPPH scavenging abilities of 43.0 ± 0.01% and 22.2 ± 0.01%, respectively. However, trypsin-hydrolysed protein had a high Fe^2+^-chelating ability, while pepsin-hydrolysed protein had high hydrogen peroxide scavenging ability. Trypsin-hydrolysed protein showed good membrane stability and inhibition of protein denaturation. The results indicated that *A. altilis* protein hydrolysates possess significant antioxidant and anti-inflammatory effects and can further lend support to food industries as functional foods.

## Introduction

Free radicals, primarily reactive oxygen species (ROS), are regularly generated during natural metabolism in the body. They can also be obtained through contaminants such as pollution, smoking, and ionising radiation and can harm cells or tissues, leading to ageing, diabetes mellitus, inflammation, cardiovascular and neurodegenerative disorders^1^. Oxidative stress, resulting from an imbalance between an enormous generation of ROS and a limited antioxidant defense system, is associated with numerous pathologies^2^. Antioxidants act by inactivating or blocking ROS generation in metabolic processes. Therefore, antioxidants are vital in the human and animal diet, as they can aid in lessening oxidative damage in the body^3^.

Inflammation is an intricate natural reaction of vascular tissues to deleterious stimuli and acts as a defensive mechanism by an organism to eliminate injurious stimuli before the start of recuperation^4^. The process is characterised by expanded endothelial porousness, penetration of leukocytes into extravascular tissues and spillage of protein-rich exudates^5^.

Developing bioactive products from food or other natural origins as food ingredients or therapeutics could help address current concerns about the potential health risks of synthetic products such as drugs^6^. As a result, there is great interest in finding new bioactive products and characterising them to identify their functional, physiological, or biological properties.

Bioactive protein hydrolysates or peptides have been isolated from different plant sources such as seeds^7^, nuts^8^, leaves^9^, and animal by-products^10^. Protein hydrolysates are obtained after protein hydrolysis by acid, alkali, enzymes and/or fermentation; however, enzymatic hydrolysis is the most common approach used in producing these bioactive ingredients^11^. The proteases trypsin and pepsin have been known to liberate bioactive protein hydrolysates from food proteins. The distinct bioactivity of food peptides to counter many diseases is dependent on their constituent peptides and structural characteristics^12^. Several studies have demonstrated the antioxidant effects of protein hydrolysates and peptides in inhibiting lipid peroxidation^13^, removing free radicals^14^ and chelating metal ions^15^.

*Artocarpus altilis* (Parkinson) Fosberg (synonym *Artocarpus communis* J.R.Forst. & G.Forst), family Moraceae, is a widely grown and nutritious tree fruit in tropical Asia, from where it was introduced to southern America and some other parts of the world. In Southwest Nigeria, *A. altilis* is referred to as *gbẹẹrẹ* and *jálókè* (meaning plucked from above/up: this is because it grows on trees and is mainly used as a substitute for yam tuber that is dug from the soil). However, it is highly unpopular due to the perishable nature of its fruits. The fruits barely last for five days after being plucked. The fruit pulp is eaten boiled, fried or pounded. *A. altilis* is abundant in vitamin C, carbohydrates, and fibre^16^. The use of breadfruit is presently restricted due to the perishable nature of its fruits. Traditionally, the bark is used as a bandage or cast to set bone fractures; the leaves are used to treat liver cirrhosis, high blood pressure, and hyperglycemia^17^. The acute toxicity of breadfruit leaves and bark revealed no mortality or toxic reaction^18^. The hexane, dichloromethane and methanol extract of the pulp had been shown to have high antioxidant activity^19^. Thus, this study is aimed at examining the antioxidant and anti-inflammatory properties of *Artocarpus altilis* fruit protein hydrolysates.

## Results

### Proximate composition of *Artocarpus altilis* fruit pulp

The percentage composition of different nutritional parameters of *A. altilis* fresh fruit, namely moisture, total ash, crude fat, crude protein, crude fibre and carbohydrate were 73.22, 1.09, 0.46, 2.99, 0.14 and 22.11%, respectively: After protein isolation, proximate analysis revealed that protein isolate (precipitate) only contained protein (68%), ash (8.8%) and moisture (23%).

### Yield of *Artocarpus altilis* fruit pulp protein hydrolysates

The percent yield of all prepared protein hydrolysates from *A. altilis* fruit pulp isolated protein is as follows: hydrolysates produced from pepsin hydrolysis at 1:8, 1:16, 1:32 enzyme: substrate ratio had 23.31, 26.25 and 37.68% yield, respectively. However, the yields of the protein hydrolysates produced after trypsin digestion at 1:8, 1:16, 1:32 enzyme: substrate ratio were 42.08, 48.30 and 48.63%, respectively.

### Physicochemical properties of *Artocarpus Altilis* fruit pulp protein hydrolysate

#### Degree of hydrolysis of *Artocarpus altilis* protein hydrolysate

**Figure 1:**
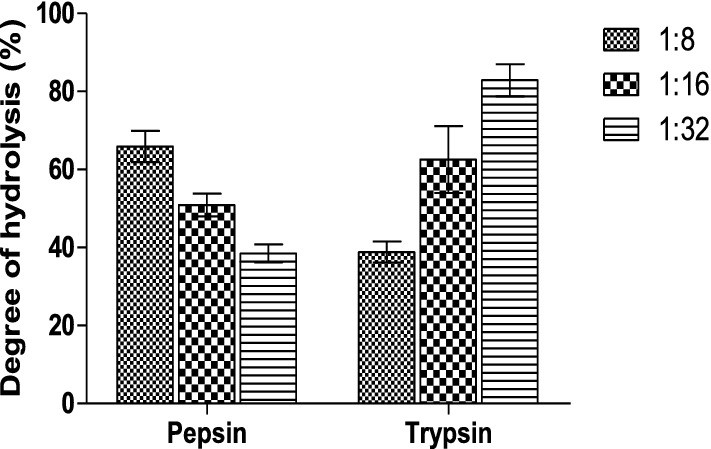
Degree of hydrolysis of *Artocarpus altilis* protein.

The DH of each AAPPH at various enzyme: substrate ratios are shown in Fig. 1. Trypsin hydrolysates exhibited an increasing degree of hydrolysis as the enzyme: substrate ratio increased; that is, 1:32 enzyme to substrate ratio had the highest degree of hydrolysis (82.84 ± 4.16%) compared to 1:8 (38.81 ± 2.71%) and 1:16 (62.58 ± 8.59%). On the other hand, pepsin hydrolysate had a decreasing degree of hydrolysis as the enzyme to substrate ratio increased; that is, 1:32 had the lowest degree of hydrolysis (38.50 ± 2.27%) compared to 1:16 and 1:8 with DH of 50.87 ± 2.94% and 65.88 ± 3.99%, respectively.

#### Amino acid (AA) composition of *Artocarpus altilis* fruit pulp isolate and protein hydrolysate

The AA composition of *A. altilis* protein isolate and trypsin hydrolysate (1:32) in g/100 g is shown in Table 1. Glutamic acid (13.57, 12.88), aspartic acid (8.98, 8.66) and leucine (9.17, 7.92) were the most abundant amino acids present in *A. altilis* protein isolate and hydrolysate, respectively, while methionine and cysteine were less abundant.

**Table 1:** Amino acid composition of *A. altilis* protein isolate and hydrolysate.

Amino acids	*A. altilis* protein isolate (g/100 g protein)	*A. altilis* protein hydrolysate (g/100 g protein)
Leucine	9.17	7.92
Lysine	4.86	4.54
Isoleucine	4.03	3.38
Phenylalanine	3.55	4.26
Tryptophan	1.87	1.39
Valine	3.09	3.60
Methionine	1.90	2.25
Histidine	2.37	2.27
Arginine	4.48	4.31
Threonine	3.06	3.31
Cystine	1.70	1.27
Alanine	3.80	3.61
Glutamic acid	13.57	12.88
Glycine	3.26	3.52
Proline	3.25	3.05
Serine	3.62	4.11
Aspartic acid	8.98	8.66
Essential amino acids	38.38	37.23
Total basic amino acids	11.71	11.12
Total acidic amino acids	22.55	21.54
Total hydrophobic amino acids	38.06	37.23

### In vitro antioxidant assays of *Artocarpus altilis* protein hydrolysates

#### DPPH radical scavenging activity

**Figure 2:**
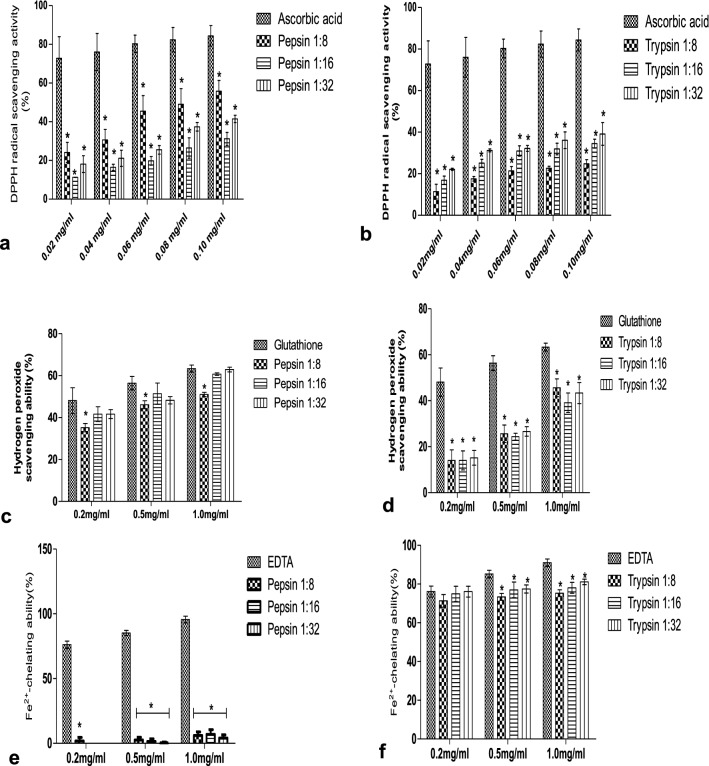
Antioxidant activities of *A. altilis* fruit pulp protein hydrolysates. DPPH radical scavenging activity (**a**) obtained with pepsin (**b**) obtained with trypsin. Hydrogen peroxide radical scavenging activity (**c**) obtained with pepsin (**d**) obtained with trypsin. Fe^2+^-chelating activity (**e**) obtained with trypsin (**f**) obtained with pepsin. Results are expressed as mean ± SD of three trials (n = 3), *represents significant difference (*p* < 0.05) with standard used.

The DPPH free radical scavenging activities (%) of AAPPH at different enzyme: substrate ratios are shown in Fig. 2(a,b). The results showed that pepsin hydrolysate at 1:8 enzyme–substrate ratio scavenged more DPPH free radicals, demonstrating the lowest EC_50_ of 0.082 mg/mL while trypsin hydrolysate at 1:8 enzyme to substrate ratio had the lowest scavenging activity (with the highest EC_50_ of 0.251 mg/mL) compared to others as shown in Table 2.

**Table 2:** EC_50_ of DPPH and hydrogen peroxide scavenging properties of *A. altilis* protein hydrolysates.

Sample	DPPH scavenging activity (EC_50_ mg/mL)	H_2_O_2_ scavenging activity (EC_50_ mg/mL)
Ascorbic acid	0.001 ± 0.00	
Glutathione		0.247 ± 0.04
Trypsin hydrolysates		
1:8	0.251 ± 0.02^a^	1.112 ± 0.13^a^
1:16	0.165 ± 0.01^a^	1.342 ± 0.09^a^
1:32	0.151 ± 0.00^a^	1.184 ± 0.25^a^
Pepsin hydrolysates		
1:8	0.082 ± 0.00^a^	0.880 ± 0.06^a^
1:16	0.176 ± 0.03^a^	0.513 ± 0.01
1:32	0.127 ± 0.01^a^	0.532 ± 0.03

All values are expressed as mean ± SD, no of trials = 3.

^a^*p* < 0.05 as compared to standard.

#### Hydrogen peroxide scavenging activity

The hydrogen peroxide scavenging activity of *A. altilis* trypsin protein hydrolysate and pepsin hydrolysate at different enzyme–substrate ratios are presented in Fig. 2c,d. The results showed that all pepsin hydrolysates had significant activity regardless of enzyme–substrate ratio, with that prepared at 1:16 enzyme–substrate ratio having the highest H_2_O_2_ scavenging activity with EC_50_ of 0.513 mg/mL of all hydrolysates. Trypsin hydrolysates, on the other hand, had lower H_2_O_2_ scavenging activity when compared with pepsin hydrolysates with EC_50_ between 1.112 and 1.342 mg/mL.

#### Iron (II)-chelating activity

The Fe^2+^-chelating activity of different concentrations of AAPPH are presented in Fig. 2e,f. *A. altilis* trypsin hydrolysate had significant Fe^2+^-chelating ability (70.13–81.88%) at various enzyme–substrate ratios regardless of the concentration. On the other hand, pepsin hydrolysate showed a very low iron chelating activity at various enzyme–substrate ratios and different concentrations.

### In vitro anti-inflammatory activities of *Artocarpus altilis* protein hydrolysates

#### Membrane stabilisation

AAPPH (0.25–1.00 mg/mL) protected the RBC membrane against lysis in a concentration-dependent manner (Table 3). Protection of RBC membrane by *A. altilis* trypsin hydrolysates (0.25–1 mg/mL) (with percent hemolysis inhibition of 88.8–93.46% at 1 mg/mL) was significantly higher (*p* < 0.05) than the standard, with trypsin hydrolysates produced at 1:16 exhibiting the highest membrane stabilization activity. On the other hand, pepsin hydrolysates also inhibited lysis, with percent hemolysis inhibition of 26.47–62.5% at 1 mg/mL, significantly lower than that of trypsin hydrolysates and the standard (acetylsalicylic acid).

**Table 3:** Percent hemolysis inhibition of *A. altilis* protein hydrolysates in human RBC membrane stabilisation test.

Sample	0.25 mg/mL	% Inhibition	0.50 mg/mL	% Inhibition	1.00 mg/mL	% Inhibition
Control	0.78 ± 0.02	–	0.78 ± 0.02	–	0.78 ± 0.02	–
ASA	0.54 ± 0.02^a^	31.28	0.33 ± 0.01^a^	57.63	0.17 ± 0.02^a^	77.80
Trypsin hydrolysates						
1:8	0.15 ± 0.01^b^	81.15	0.10 ± 0.01^b^	88.59	0.09 ± 0.01^b^	88.80
1:16	0.13 ± 0.01^b^	83.46	0.06 ± 0.01^b^	91.80	0.05 ± 0.01^b^	93.46
1:32	0.12 ± 0.01^b^	84.74	0.09 ± 0.01^b^	86.99	0.08 ± 0.01^b^	89.81
Pepsin hydrolysates						
1:8	0.63 ± 0.01^b^	19.42	0.61 ± 0.05^b^	21.79	0.57 ± 0.04^b^	26.47
1:16	0.55 ± 0.06^a^	29.36	0.37 ± 0.02^a^	52.57	0.29 ± 0.05^b^	62.50
1:32	0.70 ± 0.02^b^	10.90	0.60 ± 0.02^b^	22.69	0.42 ± 0.08^b^	46.36

All values are expressed as mean ± SD, no of trials = 3.

^a^*p* < 0.05 as compared to control.

^b^*p* < 0.05 as compared to standard (ASA—Acetylsalicylic acid).

#### Protein denaturation

AAPPH showed a concentration-dependent inhibition of protein (albumin) denaturation in a similar manner as the reference drug, acetylsalicylic acid. However, trypsin hydrolysates exhibited a significantly higher inhibition (*p* < 0.05) of protein denaturation than acetylsalicylic acid at all concentrations (0.25–1 mg/mL) (Table 4). Pepsin hydrolysates (0.25–1 mg/mL) exhibited protein denaturation but lower than that shown by trypsin hydrolysates. The protein denaturation exhibited by pepsin hydrolysates were similar to that of acetylsalicylic acid, especially at 1 mg/mL.

**Table 4:** Percent protein denaturation inhibition of *A. altilis* protein hydrolysates in egg albumin denaturation test.

Sample	0.25 mg/mL	% Inhibition	0.50 mg/mL	% Inhibition	1.00 mg/mL	% Inhibition
Control	1.27 ± 0.02	–	1.27 ± 0.02	–	1.27 ± 0.02	–
ASA	0.80 ± 0.03^a^	37.07	0.68 ± 0.01^a^	46.75	0.58 ± 0.01^a^	54.62
Trypsin hydrolysates						
1:8	0.35 ± 0.01^b^	58.68	0.53 ± 0.15^b^	70.33	0.38 ± 0.02^b^	71.58
1:16	0.42 ± 0.01^b^	67.26	0.37 ± 0.02^b^	70.68	0.26 ± 0.02^b^	79.69
1:32	0.27 ± 0.02^b^	78.87	0.17 ± 0.01^b^	86.65	0.08 ± 0.01^b^	93.82
Pepsin hydrolysates						
1:8	0.91 ± 0.01^b^	28.45	0.79 ± 0.12^b^	37.78	0.66 ± 0.04^a^	47.89
1:16	1.10 ± 0.07^b^	20.54	0.67 ± 0.03^a^	47.65	0.63 ± 0.01^a^	50.25
1:32	0.88 ± 0.01^a^	30.38	0.65 ± 0.04^a^	48.80	0.55 ± 0.01^a^	56.40

All values are expressed as mean ± SD, no of trials = 3.

^a^*p* < 0.05 as compared to control.

^b^*p* < 0.05 as compared to standard (ASA—Acetylsalicylic acid).

### Functional properties of *Artocarpus altilis* protein hydrolysate

At the end of in vitro bioactivity studies, *A. altilis* protein hydrolysed with trypsin at 1:32 had the highest activities, and its functional activities were further determined.

#### Solubility of *A. altilis* protein hydrolysate

**Figure 3:**
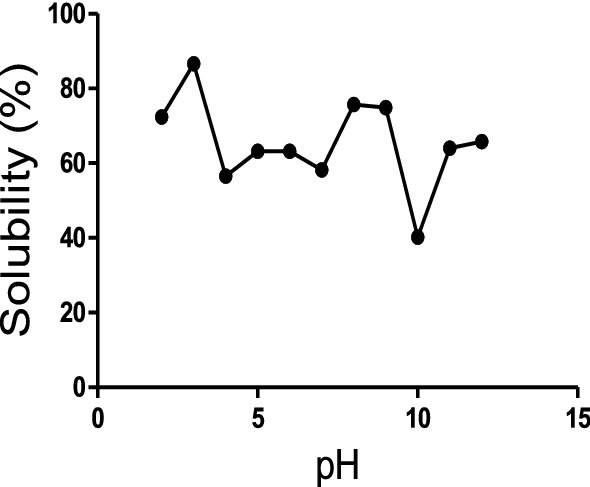
Solubility of *Artocarpus altilis* trypsin hydrolysate (1:32).

The protein solubility of *A. altilis* protein hydrolysate in the pH range of 2–12 is presented in Fig. 3. The protein hydrolysate was highly soluble over a wide pH range. The highest solubility was observed at pH 3 (86%), while the lowest solubility was obtained at pH 10 (42%).

#### Foaming properties of *A. altilis* protein isolate and hydrolysate

AAPPH showed improved foaming properties compared to the protein isolate of *A. altilis.* The protein isolate of *A. altilis* fruit did not produce an efficient foam. However, AAPPH produced from trypsin digestion produced an effective foam with 20% capacity (expansion) and 13.3% stability.

#### Emulsifying properties

Emulsifying properties of AAPPH at 0.05% and 0.1% are presented in Table 5. *Artocarpus altilis* protein hydrolysates showed higher emulsifying properties at 0.05% with 38.69 m^2^/g emulsifying activity index and 21.65 min emulsion stability index. The emulsifying properties of AAPPH were lower than those obtained for the commercial milk protein casein with emulsifying activity and emulsion stability index of 54.25% and 66.19 min, respectively.

**Table 5:** Emulsifying properties of *A. altilis* fruit pulp protein hydrolysate.

	0.05%	0.1%
EAI (m^2^/g)	38.69	35.01
ESI (min)	21.65	19.59
ESI (%)	53.81	48.95

*EAI* emulsifying activity index, *ESI* emulsion stability index.

## Discussion

Plant derived proteins and their hydrolysates are generally considered as safe functional food ingredients with various biological activities. For instance, in vitro and in vivo toxicity studies of protein hydrolysates of rapeseed and sunflower^20^, whey^21^, and green peas^22^ revealed that the protein products had no relevant toxicological effects on the various experimental models used. Also previous studies have established that various plant parts of *Artocarpus altilis* are nontoxic^18,23^.

The chemical composition of food materials impacts human health by providing nutrients essential for maintaining good health^24^. The proximate composition of breadfruit revealed that it contained an appreciable amount of carbohydrate, protein, fibre and ash. This is similar to literature reports on the composition of breadfruit pulp^25,26^.

The DH of a protein/peptide is the percentage of total peptide bonds cleaved during hydrolysis^27^. The DH of a protein reflects the amount of peptide bonds split and, therefore, the size of the resulting peptides. DH varies depending on the specific enzyme used for hydrolysis and the cleavage pattern^28^. The degree of hydrolysis of *Artocarpus altilis* fruit pulp trypsin hydrolysates was higher at 1:32 enzyme–substrate ratio (81.10%) when compared to pepsin hydrolysates obtained in this study. As enzyme concentration is increasing and substrate concentration decreasing (from 1:32 to 1:8 enzyme–substrate ratio), DH was gradually increased during pepsin hydrolysis of *A. altilis* protein, with the highest DH observed at 1:8 enzyme–substrate ratio. However, hydrolysis of *A. altilis* fruit pulp protein with trypsin showed a decreasing DH as the substrate concentration is decreased, and enzyme concentration increased. The decrease observed in DH at increasing trypsin concentration may be due to the production of inhibitory peptides during hydrolysis which brought about a reduction in enzyme activity (as reflected by low DH) at low substrate concentrations^29^. Increasing substrate concentration reduced the inhibitory effect of the inhibitory peptides, as observed by the elevated DH value at high substrate concentrations.

Proteins are an important part of the human diet, and their nutritional value relies on their AA content^30^. AA analysis is used to determine the AAs and peptides present in a sample. The results of amino acid composition in this study revealed that *Artocarpus altilis* protein and its hydrolysates are rich in aspartate, leucine, and glutamate but have low methionine and cysteine content. Essential AAs accounted for approximately 66.61% and 66.88% of the total AA content of the *A. altilis* protein isolate and hydrolysate samples, respectively, making it a moderate source of essential AA. The pulp of *Artocarpus heterophyllus* was reported to possess an adequate amount of lysine and leucine^26,31^. Gonçalves et al.^32^ also reported that the pulp of *A. altilis* contained a significant amount of aspartate and glutamate but was low in methionine and cysteine.

Different antioxidant assays allow evaluation of various aspects of oxidation. The antioxidant activities of AAPPHs varied in different antioxidant assays. DPPH radical, a stable free radical, is scavenged by hydrogen donators to become a stable diamagnetic molecule, reducing its purple colour to pale yellow^33^. DPPH radical scavenging activity of *Artocarpus altilis* pepsin hydrolysates at 1:8 enzyme–substrate was higher than other hydrolysates. Protein hydrolysates usually contain peptides or amino acids with hydrogen-donating capabilities; thereby, scavenging free radicals^34^. Studies have shown that many food-derived protein hydrolysates were capable of scavenging DPPH radicals, such as hydrolysates from wheat germ^35^, sardinelle head and viscera^36^, and fish skin^37^.

Fe^2+^-chelating activity was determined in this study to evaluate the metal-chelating ability of *Artocarpus altilis* protein hydrolysates. Ferrous ions play a great role in cell oxidant production, causing lipid peroxidation to increase^38^. Ferrozine produces a violet complex with Fe^2+^. The formation of this complex is disrupted when it comes across chelating agents, thus decreasing the intensity of the violet colour. In this study, the protein hydrolysates of *A. altilis* obtained through trypsin hydrolysis exhibited a significant chelating activity, whereas pepsin hydrolysates had little or no chelating activity. Previous studies have shown that different enzymatic hydrolysates obtained from digestion with trypsin^39^, pancreatin^40^ and alcalase^41^ showed a significant Fe^2+^-chelating ability.

Another valuable method used to determine the ability of an antioxidant to reduce pro-oxidant levels is to measure H_2_O_2_ scavenging activity^42^. The scavenging of H_2_O_2_ by antioxidants results from their electron-donating ability. At 1.0 mg/mL concentration, *A. altilis* pepsin and trypsin hydrolysates showed excellent H_2_O_2_ scavenging activity. Pepsin hydrolysates recorded nearly 65% scavenging activity, and trypsin hydrolysates around 43%. H_2_O_2_ is not very reactive, but it can easily be changed into better reactive species such as ^1^O2 and ·OH radicals, causing lipid peroxidation or harmful consequences in cells^43^.

An in vitro evaluation of anti-inflammatory properties by human red blood cells (HRBC) was chosen because of the erythrocyte membrane’s similarity to the lysosomal membrane^44^. Thus, stabilisation of the RBC by protein hydrolysates may suggest that it may also stabilise lysosomal membranes. A stabilised lysosomal membrane is critical for limiting inflammatory response by preventing lysosomal components of activated neutrophils, such as bactericidal enzymes and proteases, from leaking into the extracellular space and causing further tissue inflammation^45^. In this study, *A. altilis* trypsin hydrolysates produced significant anti-hemolytic activity on HRBC membranes at all concentrations tested (0.25–1.00 mg/ml), indicating strong stabilising effects on hypotonicity-induced HRBC membranes and potent anti-inflammatory effects. The activity of pepsin, trypsin and alcalase protein hydrolysates of a gastropod *Harpa ventricosa* had earlier been reported to protect the red blood cell, with trypsin hydrolysates showing the highest percentage inhibition^46^.

It has been reported that many non-steroidal anti-inflammatory drugs (NSAIDs) can stabilise heat-processed albumin (prevent denaturation) at physiological pH levels (6.2 to 6.5)^47^. Several inflammatory arthritic diseases, such as rheumatoid arthritis are induced by altering the hydrophobic, electrostatic, hydrogen, and disulfide bonds of tissue proteins, thus leading to auto-antigens^48^. In the present study, *A. altilis* protein hydrolysate inhibited the denaturation of proteins; thus, it can control auto-antigen production. In summary, *A. altilis* protein hydrolysates comparably showed considerable anti-inflammatory activity to the standard drug, acetylsalicylic acid.

From the in vitro bioactivities studies of *A. altilis* protein hydrolysates, trypsin hydrolysate obtained at 1:32 enzyme–substrate ratio was the most effective, and its functional properties were further elucidated. Concerning their potential industrial applications, solubility as a function of pH is an essential characteristic of protein hydrolysates. Generally, protein solubility is affected by the hydrophobic (protein–protein) or hydrophilic (protein-solvent) interactions within their surface. Protein solubility in food is a hydrophilic property because the solvent is usually water^49^. Peptides produced by different enzymes may have different sizes and charges, affecting their solubility and other properties^50^. *Artocarpus altilis* trypsin hydrolysate was soluble across pH 2–12 but primarily soluble at pH 3 and least soluble at pH 10. This can be attributed to the fact that hydrolysed peptides with a high DH usually contain more polar residues and have low molecular weights, which enhance the number of hydrogen bonds with water, increasing the solubilisation compared to hydrolysed peptides with a low degree of hydrolysis^51^.

In order to foam effectively, a protein must have the ability of rapidly migrating to the air–water interface, unfolding, and readjusting at the interface^52^. The stability of foam is dependent on the nature of the foam and the degree to which protein–protein interactions occur within the matrix^53^. Flexible protein domains are believed to increase aqueous viscosity, protein concentration, and foam thickness^54^. *A. altilis* protein isolate had zero foaming expansion and stability, whereas the trypsin hydrolysate had good expansion and stability. The poor foaming expansion of the protein isolate could be because of globular structure of the protein, which prevents them from forming interfacial membranes around the air bubbles. Enzymatic hydrolysis reduces molecular weight and increases flexibility of proteins, facilitating the formation of interfacial membranes and foams^55^. Several protein hydrolysates have been reported with good foam expansion and stability^34,50^.

Emulsification is one of the factors considered in the food industry. Emulsification causes molecules to quickly attach to freshly formed droplets, reducing the possibility of interfacial tension. A higher proportion of nonpolar groups in hydrolysed proteins contributes to their emulsifying ability^56^. The EAI of AAPPH decreased with increasing concentration, and there was a decrease in ESI as concentration increased up to 0.1%. This suggests that the protein hydrolysate AAPPH contained peptide chains, producing stronger protein–protein interactions that favour emulsion formation. Kinsella and Melachouris^57^ stated that the relationship between emulsifying activity and protein concentration is explained by adsorption kinetics. The adsorption of protein at the oil–water interface is diffusion-controlled at low protein concentrations. When protein concentrations are high, the activation energy barrier counters the migration of protein diffusion-dependent^58^, decreasing EAI and ESI. However, the emulsifying properties of AAPPH were lower than those obtained for casein, a milk protein used as an emulsifying agent in food formulations and production of materials such as emulsion paints.

## Conclusions

At the end of this study, *Artocarpus altilis* protein hydrolysates produced from trypsin and pepsin digestion had significant bioactivities (antioxidant and anti-inflammatory activities). However, those obtained with trypsin had the highest in vitro antioxidant activity (able to scavenge free radicals effectively and chelate Fe^2+^) and anti-inflammatory activities (inhibited red blood cell lysis and protein denaturation efficiently) at 1:32 enzyme–substrate ratio. The hydrolysate also exhibited good functional properties. As a result, *A*. *altilis* protein hydrolysates (especially those obtained with trypsin) have potential application as a functional food ingredient and therapeutic tool in managing diseases associated with oxidative stress and inflammation.

## Materials

The enzymes trypsin (2000 U/g) and pepsin (> 250 U/g), bovine serum albumin (BSA), bovine casein, gluthathione, ethylene diaminetetraacetic acid (EDTA), 1,1-diphenyl-1-picrylhydrazyl (DPPH)were purchased from Sigma Aldrich (St. Louis, MO, USA). All other reagents were purchased from Loba Chemie (Mumbai, India).

## Methods

All methods were carried out in accordance with relevant guidelines and regulations. All experimental protocols in this study were approved by Afe Babalola University, Ado-Ekiti, Nigeria (ABUAD) Scientific Research Committee.

The use of a hen’s egg (1–8 h old) in this study was approved by the Institutional Animal Ethics Committee of Afe Babalola University, Nigeria (Approval number: 22/ABUAD/SCI/024). The methods were carried out according to the institutional operating procedures for the use of animals and their products, following NIH guidelines, and reported in accordance with ARRIVE guidelines.

Informed consent was obtained for the membrane stabilisation study from the participant.

### Preparation of *Artocarpus altilis* fruit

Matured *A. altilis* fruits were collected from Ilode farmland along Ifewara road, Ile-Ife, Nigeria (coordinates: 7.4809° N, 4.5663° E). The plant was identified and authenticated by Mr. G.A. Ademoriyo at the IFE Herbarium, Department of Botany, Obafemi Awolowo University, Ile-Ife, where a voucher copy was deposited and voucher number: IFE-18148 assigned. The collection of *A. altilis* plant and concurrent experimental research was carried out following the guidelines and legislation described in Zhang and WHO^59^.

### Proximate composition of *Artocarpus altilis* fresh fruit pulp

The proximate composition, including crude protein, crude fibre, ash, moisture and fat contents, of the fresh fruit pulp were done according to the method of AOAC^60^.

### Preparation of protein isolates of *Artocarpus altilis* fruit pulp

*Artocarpus altilis* fruits were peeled, and the pulps were diced. The diced pulp was blended in 0.1 M NaOH (1:5 w/v) and stirred for 4 h at 4 °C. This mixture was centrifuged at 4000 rpm for 20 min. The supernatant was subjected to acid precipitation using 6 M HCl to pH 4.0 and further centrifuged at 3500 rpm for 15 min. The supernatant was discarded, and the pellet was collected, lyophilised, and stored at − 4 °C as protein isolate.

### Preparation of *A. altilis* protein hydrolysates

*A. altilis* protein isolate was digested separately using pepsin and trypsin at optimum conditions as described by Osukoya et al.^50^. Briefly, *A. altilis* protein isolates (4 mg/mL) were separately hydrolysed with the enzymes pepsin and trypsin (1 mg/mL) in 0.1 M glycine–HCl buffer, pH 2 and 0.1 M phosphate buffer, pH 8, respectively, while varying enzyme: substrate ratio at 1:8, 1:16 and 1:32 (v/v) (approximately 1:32, 1:64 and 1:128 w/w, respectively). The mixtures were incubated for 4 h at 37 °C with incessant mixing at intervals, and the pH was monitored throughout hydrolysis. At the end of the 4th hour, the mixtures were boiled at 100 °C for 10 min to terminate the reaction, promptly placed on ice, and centrifuged at 4000 rpm for 20 min. The supernatants were collected, freeze-dried, and stored at − 20 °C.

### Determination of protein concentration

The protein concentrations of *A. altilis* protein isolates and protein hydrolysates were determined using the Lowry’s method as described by Olson and Markwell^61^ using 1 mg/mL bovine serum albumin (BSA) as protein standard. Absorbance was taken at 660 nm.

### Yield of the protein hydrolysates

The yield (%) of each protein hydrolysates was calculated as the ratio of total nitrogen in each hydrolysates and the total nitrogen in *A. altilis* protein isolates as a percentage.

$${\text{Yield }}\left( \% \right) \, = \frac{{{\text{Total }}\,{\text{nitrogen}}\,{\text{ in }}\,{\text{protein }}\,{\text{hydrolysates}}}}{{{\text{Total}}\,{\text{ nitrogen}}\,{\text{ in }}\,{\text{protein}}\,{\text{ isolate}}}} \times 100$$

### Physicochemical properties of the protein hydrolysates

#### Degree of hydrolysis

The degree of hydrolysis (DH) of *A. altilis* hydrolysed protein was determined using the trichloroacetic acid (TCA) method as described by Osukoya et al.^50^. Briefly, an equal volume of trichloroacetic acid (20%) was added to *A. altilis* protein hydrolysates and kept at 4 °C for 30 min. The mixture was centrifuged at 3000 rpm for 10 min and the 10% TCA-soluble peptides obtained were assayed using the Lowry’s method. The percentage DH was calculated as follows:

$$ {\text{DH }}\left( \% \right) \, = \frac{{10{\text{\% TCA}} - {\text{soluble}}\,{\text{ peptides}}}}{{{\text{Total}}\,{\text{ protein}}\,{\text{ content}}}} \times 100 $$

#### Amino acid composition

Amino acid (AA) analysis was determined as described by AOAC^62^ with modifications. *A. altilis* protein isolate, and protein hydrolysate were dried, hydrolysed, evaporated and applied to the Applied Biosystems PTH Amino Acid Analyzer.

### In vitro antioxidant activities of *Artocarpus altilis* protein hydrolysates

#### 2,2-diphenyl-1-picrylhydrazyl (DPPH) free radical scavenging assay

The scavenging activity of AAPPHs was determined by the stable radical DPPH method described by Zhu et al.^63^. An aliquot of 100 μL of varying concentrations of AAPPH (0.02–0.1 mg/mL) was mixed with 100 μL DPPH (0.25 mM in 85% methanol) in a 96-well microtitre plate, and incubated for 30 min in the dark. The absorbance was taken at 520 nm using a plate reader. The blank contained methanol in place of AAPPH, and ascorbic acid (0.02–0.1 mg/mL) was used as standard. The percentage inhibition of the standard and sample was calculated as follows:

$$\% {\text{ Inhibition }} = \frac{Absorbance\, of\, blank - Absorbance \,of\, sample}{{Absorbance \,of\, blank}} \times 100$$

#### Hydrogen peroxide (H_2_O_2_) scavenging activity assay

H_2_O_2_ scavenging activity of AAPPHs was determined according to the method of Zhang et al.^64^ with minor modification. Varying concentrations of AAPPH (0.2–1.0 mg/mL; 1000 μL) were mixed with 2000 μL of H_2_O_2_ (20 mM in phosphate buffered saline (PBS), pH 7.2) and incubated for 10 min. Absorbance was taken at 230 nm. Glutathione was used as a standard, and the blank solution contained PBS and H_2_O_2_. The percentage inhibition of the standard and sample was calculated as follows:

$$\% {\text{ Inhibition }} = \frac{Absorbance \,of\, blank - Absorbance \,of\, sample}{{Absorbance \,of\, blank}} \times 100$$

#### Iron (Fe^2+^)-chelating ability assay

The in vitro Fe^2+^-chelating ability of AAPPH was determined as described by Osukoya et al.^50^. FeSO_4_ (500 μM, 900 µL) and varying concentrations of AAPPH (0.2–1.0 mg/mL; 150 µL) were mixed and left for 5 min. This was followed by adding 78 µL phenanthroline (0.2% in ethanol), and absorbance was immediately read at 510 nm. EDTA (0.2–1.0 mg/mL) was used as a standard, while the blank contained distilled water. The percentage chelation was calculated as follows:

$$\% {\text{ Chelation }} = \frac{Absorbance\, of\, blank - Absorbance\, of\, sample}{{Absorbance\, of\, blank}} \times 100$$

### Evaluation of in vitro anti-inflammatory activity

#### Membrane stabilisation method

The method described by Parvin et al. ^65^ was followed. Briefly, human blood (10 mL) was obtained from a willing and healthy volunteer. The blood was mixed with 10 mL of Alsever solution (2.05% *D*-glucose, 0.8% sodium citrate, 0.055% citric acid, 0.42% NaCl) and centrifuged at 3000 rpm at room temperature for 15 min. The supernatant was discarded, the packed red blood cell was washed with isosaline solution, and a 4% suspension in isosaline solution was prepared. A 1000 μL of 0.1 M phosphate buffer pH 8.0, 2000 μL of hyposaline (0.36% NaCl), and 500 μL of red blood cell suspension were mixed with varying concentrations of AAPPH (0.25–1.0 mg/mL). Distilled water was used instead of AAPPH as blank and acetylsalicylic acid (0.25–1.0 mg/mL) as standard. The mixture was incubated for 30 min at 37 °C and centrifuged for 20 min at 3000 rpm. The haemoglobin content of the supernatant was measured at 560 nm, and the percentage inhibition was calculated as follows:

$$\% {\text{ Inhibition }} = \frac{Absorbance\, of\, blank - Absorbance\, of \,sample}{{Absorbance\, of\, blank}} \times 100$$

#### Protein denaturation method

Inhibition of protein denaturation assay was done as described by Ullah et al.^66^. A 100 μL of albumin from a hen’s fresh egg (1–8 h) was mixed with 2800 μL of PBS, pH 6.4 and 2000 μL of varying concentrations of AAPPH (0.25–1.0 mg/mL), incubated for 15 min at 37 °C and boiled for 5 min at 70 °C. The absorbance was measured at 660 nm. Acetylsalicylic acid (0.25–1.0 mg/mL) was used as a reference drug. The blank contained PBS instead of AAPPH, and the percentage inhibition of protein denaturation was calculated thus:

$$\% {\text{ Inhibition }} = \frac{Absorbance\, of\, blank - Absorbance \,of\, sample}{{Absorbance \,of \,blank}} \times 100$$

### Functional properties of *Artocarpus altilis* protein hydrolysate

The protein solubility, foaming and emulsifying properties of AAPPH with overall maximal in vitro antioxidant and anti-inflammatory activities were further determined.

#### Protein solubility of *Artocarpus altilis* protein hydrolysate

AAPPH was dissolved in distilled water (1:4 w/v) and stirred continuously. The pH of the solution was adjusted to 2–12 with 6 M HCl or 6 M NaOH, stirred for 25 min and centrifuged at 3000 rpm. The peptide content of the supernatant was determined using Lowry’s method, and solubility (%) was estimated as follows:

$${\text{Solubility }}\left( {\text{\% }} \right) = \frac{{{\text{Peptide }}\,{\text{content}}\,{\text{ in}}\,{\text{ supernatant}}}}{{{\text{Peptide}}\,{\text{ content}}\, {\text{in}}\,{\text{ protein }}\,{\text{hydrolysate}} }} \times 100$$

#### Foaming properties of *Artocarpus altilis* protein hydrolysate

AAPPH (0.15 g) was suspended in 15 mL distilled water, stirred for 1 h at 30 °C and pH adjusted to 7. The mixture was transferred into a measuring cylinder and the volume (V_o_) was taken. The mixture was further homogenized for 30 s and instantly transferred into a measuring cylinder and volume (V_T_) taken. The mixture was left at room temperature and volume V_30_ was taken after 30. The foam expansion and stability were determined according to Osukoya, et al.^50^ as follows:

$${\text{Foaming}}\,{\text{ expansion }}\left( {\text{\% }} \right) = \frac{{{\text{V}}_{T} - V_{o} }}{{V_{o} }} \times 100$$

$${\text{Foaming }}\,{\text{stability }}\left( {\text{\% }} \right) = \frac{{{\text{V}}_{30} - V_{o} }}{{V_{o} }} \times 100$$

#### Emulsifying properties of *Artocarpus altilis* protein hydrolysate

The emulsifying properties of AAPPH, including emulsifying activity index (EAI) and emulsion stability index (ESI), were determined according to the procedure of Noman et al.^67^. Six millimetres of AAPPH (0.5% and 1%) and 10 mL of olein oil were mixed and homogenised for 2 min at 2000 rpm. A 50 μL amount was taken from the bottom of the emulsion formed and mixed with 0.1% sodium dodecyl sulfate (500 µL). The absorbance of the solution was measured after 0 and 10 min at 500 nm. Casein was used as a reference emulsifier. Emulsifying properties were calculated thus:

$${\text{EAI }}\left( {{\text{m}}^{{2}} /{\text{g}}} \right) \, = \, {{\left( {{2 } \times { 2}.{3}0{3} \times {\text{ A }} \times {\text{ DF}}} \right)} \mathord{\left/ {\vphantom {{\left( {{2 } \times { 2}.{3}0{3} \times {\text{ A }} \times {\text{ DF}}} \right)} {\left( {{\text{I}}\upphi {\text{C}}} \right)}}} \right. \kern-0pt} {\left( {{\text{I}}\upphi {\text{C}}} \right)}} \,$$

$${\text{ESI }}\left( {{\text{min}}} \right) \, = \, {{\left( {{\text{A}}_{0} \times { 1}0} \right)} \mathord{\left/ {\vphantom {{\left( {{\text{A}}_{0} \times { 1}0} \right)} {\left( {{\text{A}}_{0} {-}{\text{ A}}_{{{1}0}} } \right)}}} \right. \kern-0pt} {\left( {{\text{A}}_{0} {-}{\text{ A}}_{{{1}0}} } \right)}}$$

$${\text{ESI }}\left( \% \right) \, = { 1}00 - \left( {{\text{A}}_{0} {-}{\text{ A}}_{{{1}0}} /{\text{A}}_{0} } \right) \, \times { 1}00$$

where A = Absorbance at 500 nm; DF = dilution factor; I = path length of cuvette (m); Ф = oil volume fraction (0.25); C = protein concentration in aqueous phase (g/m^3^); A_0_ = Absorbance at 0 min; A_10_ = Absorbance at 10 min.

### Statistical analysis

The 50% effective concentration (EC_50_) was determined from a linear regression analysis on Microsoft Excel 2019 (version 2210).

Results were expressed as mean ± standard deviation and subjected to statistical analysis using one-way analysis of variance (ANOVA), which was followed by Tukey post hoc test to determine significant differences between means. Differences were considered significant with a *p* < 0.05. The graphs and statistical analyses were performed in GraphPad Prism software.

### Ethics approval and consent to participate

All experiments involving human participants were done in accordance with the ethical guidelines of Afe Babalola University, Ado-Ekiti, Nigeria (ABUAD) Health Research Ethics Committee (Approval Number: ABUADHREC27/04/2022/06) and with the 1964 declaration of Helsinki (and later amendments). The researcher’s blood was used for the membrane stabilisation test. No external participant was used in the study. Informed consent was obtained from the researcher from whom blood was obtained.

## Data Availability

All data generated or analysed during this study are included in this published article.
